# *Caryocar brasiliense* Cambess. Pulp Oil Supplementation Reduces Total Cholesterol, LDL-c, and Non-HDL-c in Animals

**DOI:** 10.3390/molecules25194530

**Published:** 2020-10-03

**Authors:** Gabriela Torres Silva, Carolina Di Pietro Fernandes, Priscila Aiko Hiane, Karine de Cássia Freitas, Priscila Silva Figueiredo, Aline Carla Inada, Wander Fernando Filiú, Iriani Rodrigues Maldonade, Ângela Alves Nunes, Lincoln Carlos Silva de Oliveira, Anderson Rodrigues Lima Caires, Flavio Michels, Camila Jordão Candido, Leandro Fontoura Cavalheiro, Marcel Arakaki Asato, Juliana Rodrigues Donadon, Bernardo Bacelar de Faria, Mariana Bento Tatara, Julio Henrique Rosa Croda, Arnildo Pott, Carlos Eduardo Domingues Nazário, Rita de Cássia Avellaneda Guimarães

**Affiliations:** 1 Program in Health and Development in the Midwest Region, Medical School, Federal University of Mato Grosso do Sul, Campo Grande 79070-900, Brazil; gabitorres483@gmail.com (G.T.S.); priscila.hiane@ufms.br (P.A.H.); kcfreitas@gmail.com (K.d.C.F.); pri.figueiredo92@gmail.com (P.S.F.); inadaaline@gmail.com (A.C.I.); cahjordao@gmail.com (C.J.C.); julianadonadon@yahoo.com.br (J.R.D.); 2 Pharmaceutical Science, Food and Nutrition Faculty, Federal University of Mato Grosso do Sul-UFMS, Campo Grande 79079-900, Brazil; carolfernands2@gmail.com (C.D.P.F.); wander.filiu@gmail.com (W.F.F.); arnildo.pott@gmail.com (A.P.); 3 Laboratory of Food Science and Technology, Brazilian Agricultural Research Corporation (Embrapa Hortaliças), Brasília 70275-970, Brazil; iriani.maldonade@embrapa.br; 4 Campo Grande University, Campo Grande 79005-000, Brazil; nunysnutri@yahoo.com.br; 5 Chemistry Institute, Federal University of Mato Grosso do Sul, Campo Grande 79070-900, Brazil; lincoln.cso@gmail.com (L.C.S.d.O.); lernfc@gmail.com (L.F.C.); cenazario@gmail.com (C.E.D.N.); 6 Optics and Photonics Group, Institute of Physics, Federal University of Mato Grosso do Sul, Campo Grande 79070-900, Brazil; andercaires@gmail.com (A.R.L.C.); flavio.michels@ufms.br (F.M.); 7 Medical School, Federal University of Mato Grosso do Sul, Campo Grande 79070-900, Brazil; marcel_arakakiasato@hotmail.com; 8 Diagnostic Medicine Laboratory-Scapulatempo, Campo Grande 79002-17, Brazil; bacelarfaria@gmail.com; 9 Health Science Research Laboratory, Federal University of Grande Dourados, Dourados 79804-970, Brazil; marianabtatara@gmail.com (M.B.T.); juliocroda@gmail.com (J.H.R.C.); 10 School of Medicine Federal University of Mato Grosso do Sul, Oswaldo Cruz Foundation—Fiocruz, Campo Grande 79074-460, Brazil

**Keywords:** fatty acids, natural products, pequi

## Abstract

The fruit of *Caryocar brasiliense* Cambess. is a source of oil with active compounds that are protective to the organism. In our work, we analyzed the physicochemical characteristics and evaluated the effects of supplementation with *C. brasiliense* oil in an animal model. We characterized the oil by indices of quality and identity, optical techniques of absorption spectroscopy in the UV–Vis region and fluorescence, and thermogravimetry/derived thermogravimetry (TG/DTG). For the animal experiment, we utilized mice (*Mus musculus*) supplemented with lipidic source in different dosages. The results demonstrated that *C. brasiliense* oil is an alternative source for human consumption and presents excellent oxidative stability. Primarily, it exhibited oleic MFA (53.56%) and palmitic SFA (37.78%). The oil level of tocopherols and tocotrienols was superior to the carotenoids. The supplementation with *C. brasiliense* oil reduced the levels of total cholesterol, LDL-c, and non-HDL-c. Regarding visceral fats and adiposity index, the treatment synergically supplemented with olive oil and *C. brasiliense* oil (OO + CO) obtained the best result. Therefore, *C. brasiliense* oil is a high quality product for consumption. Its supplementation promotes beneficial effects mainly on the lipidic profile.

## 1. Introduction

Vegetable oils are valuable natural source of triacylglycerols (TAG), which are composed of fatty acids (FAs) and glycerol. The chain length of FA can vary between C:8 and C:24, wherein the most prevalent are those with C:16 and C:18 [1]. The characteristics of edible oils and fats are subject to several processing steps, mainly during extraction [2].

The chemical composition also has an influence on oil quality, which shall be fully determined herein, considering the involved industrial process, together with its resulting oxidative stability [3]. Besides, aspects of color, values of acidity, peroxides, and others shall be analyzed, complementing and proving its composition as an oil to be considered adequate for human consumption [4].

There is growing evidence that FAs play a crucial role in human nutrition [5], including the therapeutic and prophylactic prevention of diseases [6]. Therefore, it is necessary to investigate the physicochemical properties of edible oils, to know their characterization, and the behavior of their compounds, so that their pathway of action in the organism is understood [7].

FAs have distinct effects on cell stress, and evidence indicates that excess consumption of saturated fatty acid (SFA) has detrimental effects on health, favoring the inflammatory process [8], compared to monounsaturated fatty acids (MUFAs) and polyunsaturated (PUFAs) [9]. The most abundant FAs in the organism, considering both the adipose tissue reservoirs and the dietary fat intake, are oleic (MUFA) and palmitic (SFA) free fatty acids. [10].

The oleic and palmitic FAs are predominant components of olive oil [11], commonly utilized in its extra virgin form as the primary source of fat in the Mediterranean diet, for its high content of MUFAs and polyphenols [12,13]. Their ingestion acts as an effective method to modulate factors related to oxidative stress and inflammation through the biomarkers CRP and IL-6 [14], besides improving the lipidic profile reducing hyperlipidemia in the vascular system [15].

Among the main oils rich in MUFAs is soybean oil. This oil is highly produced and heavily used oil in cooking, processed foods, margarines, and is the oil of choice in many restaurants [16]. In a study by Deol et al. (2015), mice fed a high soybean oil diet showed obesogenic and diabetogenic effects when compared to other lipid sources [17].

Although not being one of the main traditional oil crops, such as soybean, canola, sunflower and [18], another vegetable oil with a high content of MUFAs is *C. brasiliense*, with 54.28% oleic FA [19,20]. It is from a typical Brazilian fruit known as pequi, with a yellow-orangish colour and peculiar odor, reported as being rich in antioxidant compounds, such as phenolics, carotenoids, tocopherols, phytosterols [21], lycopene, and beta-carotene [22], besides showing anti-inflammatory and cardioprotective effect [23].

Hildebrand et al. (2017) highlight the importance of new studies that evaluate foods with protective effects on human health, especially those that have anti-inflammatory action [24]. Therefore, the main objective of our study was to analyze the physical and chemical characteristics of the pulp oil of *Caryocar brasiliense* Cambess., to evaluate the effects of supplementing this oil and other lipid sources, such as soybean oil and olive oil in a model animal.

## 2. Results and Discussion

The indices of acidity, peroxides, saponification, and iodine are some of the main parameters that indicate oil quality [25] and together are also correlated to product stability [9]. The acidity index indicates the presence of free fatty acids that, in significant quantities, turn the oil more liable to rancification [25]. In our study, the acidity content found in the *C. brasiliense* oil (Table 1) is within the maximum limit allowed for crude oils (<4 mg KOH/g) [4]. Similarly to our study, values within the recommended acidity standard were obtained (0.68 mg KOH/g^−1^) in another species, *Caryocar coriaceum* Wittm. [26], and also in *Acrocomia aculeata* oil (0.97 mg KOH/g^−1^), composed of a profile of FAs similar to *C. brasiliense* oil [27].

The peroxide index is an indicator of the initial stages of rancification and a measure of primary products of the lipidic oxidation [25]. The peroxide index presented a high value (13.63 mEq O_2_ kg^−1^) that can be explained by the oil unsaturation degree, which may indicate the start of the oxidation process. However, the index is following the maximum standard for crude oils (<15 mEq O_2_ kg^−1^), demonstrating adequacy for consumption [4]. Differing from the index of *C. brasiliense*, another study showed a result under 4.40 mEq O_2_ kg^−1^, found in *C. coriaceum* oil [28].

The iodine index is a measure of the unsaturation of fats and oils and consequentially the susceptibility to oxidation [29]. The obtained iodine index (76.7 I_2_/100^−1^ g) is close to values found in analyses of 10 oils of different cultivars of olive (*Olea europaea* L.), between 80 and 89 I_2_/100^−1^ g [30]. Its counterpart, the oil of the fruit of *Byrsonima cydoniifolia* A. Juss., also native to South America, has an iodine index with significantly higher unsaturation degree (120.84 I_2_/100^−1^ g) [31], compared with *C. brasiliense* oil. This indicates higher stability of *C. brasiliense* compared with the others cited; the the higher the unsaturation, the lower its oxidative stability [25].

The saponification index is related to its molecular weight or chain length of triglycerides that compose the oil [32]. The reported result presented a low value (136.5 mg KOH g^−1^), near the recommended level for olive oil (184–196 mg KOH/g) and palm oil (190–209 mg KOH/g) [4,33], with characteristics similar to *C. brasiliense*. This indicates that the oil does not contain many fatty acids with low molecular weight.

Another parameter for oil characterization is it coloration, one of the initial factors pointing to possible lipidic oxidation and consequent degradation, as color alteration is caused by the degradation of essential FAs and others compounds [2]. The fruit of *C. brasiliense* is considered a source of carotenoids [23]; the liposoluble pigments responsible for the orange and red coloration [34].

The analyzed *C. brasiliense* oil evidenced the presence of carotenoids, due to the positive values of a* and b* (Table 1), showing that most pigments are yellowish, followed by red, which is correlated with the total content of carotenoids detected in the samples. The high value of C* indicates a high-intensity color of the oil, thus considered dark. High contents of carotenoids are found in a limited number of edible oils, when compared with *Mauritia flexuosa* oil and palm oil, considered rich in this compound, with concentrations of 1722.87 mg kg^−1^ [35] and 1385 mg kg^−1^, respectively [36]. *C. brasiliense* oil stands out, with a content of 2.39 µg/g of total carotenoids.

**Figure 1:**
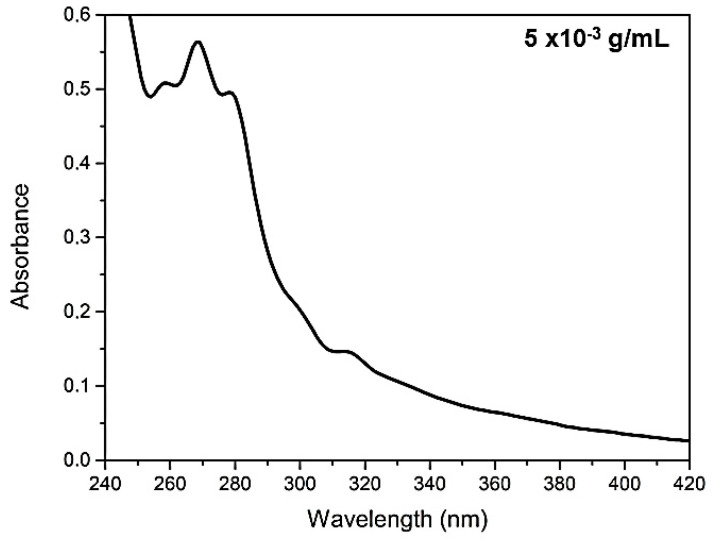
Molecular absorption spectra of *Caryocar brasiliense* oil, obtained at 200–600 nm.

**Figure 2:**
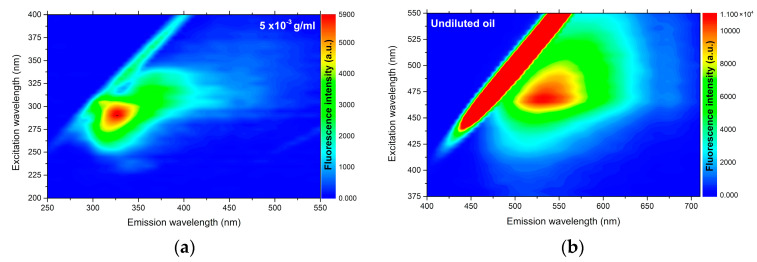
Excitation–emission map of *Caryocar brasiliense* oil obtained by exciting between 200 and 400 nm and in the emission 250 and 600 nm range. (**a**) *Caryocar brasiliense* oil at 5 × 10^−3^ g mL^−1^ concentration; (**b**) Undiluted *Caryocar brasiliense* oil.

In Figure 1 it is possible to observe the analysis of UV–Vis absorption and in Figure 2 the fluorescence of *C. brasiliense* oil at the concentration of 5 × 10^−3^ g mL^−1^ (a) and the result of fluorescence in pure oil (b). We verified the presence of natural antioxidants, such as tocopherols, and tocotrienols, named vitamin E [37] by the absorption band approximately in 313 nm (Figure 1) and the emission band in 327 nm, excited in 290 nm (Figure 2a).

With regard to the fluorescence analysis directly in undiluted vegetable oil (2b), we observed the presence of an emission band centered at 530 nm when excited at 470 nm. That fluorescence band can be attributed to the carotenoids [38]. The presence of chloropylls is usually emitted in the range of 650 to 750 nm [7,37], and was slightly ranked in the analysis of undiluted oil (Figure 2b). That datum is correlated with the value of total carotenoids found (Table 1), demonstrating the low concentration of carotenoids and higher presence of tocopherols *C. brasiliense* oil, with α-tocopherol standing out.

**Figure 3:**
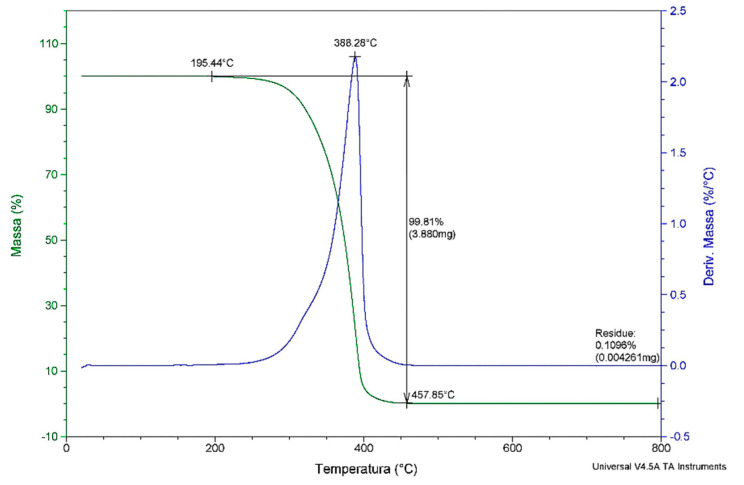
Thermogravimetry/derivate thermogravimetry (TGA/DTG). Curves of Caryocar brasiliense oil.

The thermal degradation (Figure 3) of *C. brasiliense* oil was observed mainly in the range of 195 °C to 457 °C, later stabilizing its mass. The peak occurred at 388 °C, with a loss of total mass of 99.8%. The residues were 0.1%, below possible analytical errors. The loss of initial mass at ~195 °C can be attributed to moisture loss of the oil and the volatilization of compounds such as aldehydes and short-chain fatty acids, a common factor in vegetable oils, as the oil composition influences the total mass loss [39].

The chemical composition of *C. brasiliense* oil includes antioxidant and oxidizable compounds that influence its oxidative stability [40]. The role of α-tocopherol is not yet totally defined, but it is known that its presence improves the oxidative stability in vegetable oils [3]. Moreover, it is considered one of the best phenolic antioxidants as it rapidly reacts with the alkyl peroxyl radical, forming more stable adducts and protecting lipids from peroxidation [41].

**Figure 4:**
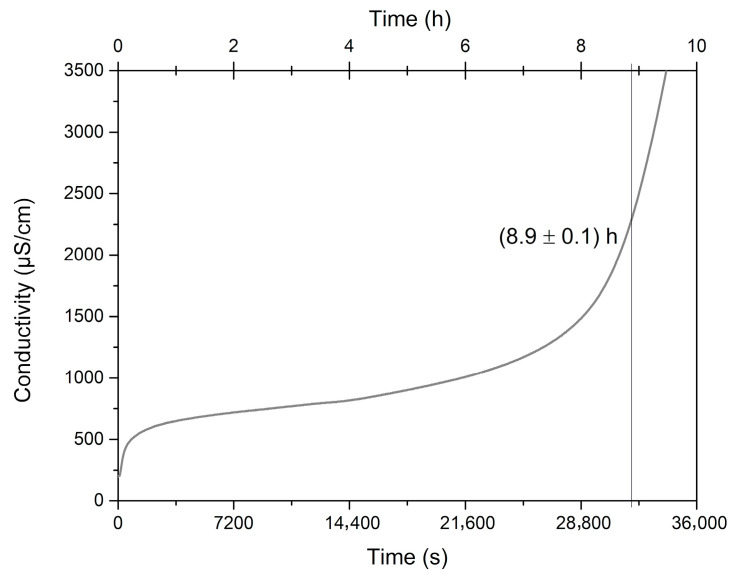
Electrical conductivity versus time determined by the Rancimat method in *Caryocar brasiliense* oil.

There is no minimum period of induction recommended for good quality edible oils; nevertheless, we consider that the *C. brasiliense* oil reached a long induction period of 8.6 h and consequent excellent oxidative stability (Figure 4), mainly because of the high presence of SFA and MFA and other minor components. This means *C. brasiliense* oil has a suitable shelf life. Similarly, the *C. brasiliense* pulp and nut oils evaluated by Torres et al. [21] differed in time of oxidative induction between 7.33 and 15.91 h. Other oils considered very stable present induction values of 12 h (soybean), 9.96 h (maize), and 8.63 h (canola) [3].

Moreover, we can consider that the prevention mechanism of oxidation through antioxidants occurred in the *C. brasiliense* pulp oil as its content of unsaturated FAs is higher than saturated (Table 2), which make the oil more susceptible to degradation [42,43]. The characterization of the profile of the FAs of *C. brasiliense* oil revealed a high content of monounsaturated fatty acids (MUFA), especially the oleic FA (56.61%), followed by the saturated FA palmitic (37.78%) and polyunsaturated linoleic acid (3.9%). The values we found are similar to those reported by Nascimento-Silva et al. (2019) [23]: 55.87%, 35.17%, and 1.53%, respectively, except linoleic FA that has a higher percentage in their study. Similar content (1.36%) was also reported by Roll et al. (2018) [19].

The oxidative stability, the parameters of quality within recommendations for edible vegetable oils, and the prevalence of unsaturated FAs are the factors that determine whether an oil is adequate for human consumption. Moreover, oils with a high level of MUFAs and PUFAs can improve the levels of serum lipids [44]. After 90 days of supplementation, we observed better levels of total cholesterol, LDL-c, and non-HDL-c (Table 3) in the groups supplemented with *C. brasiliense* oil as a lipidic source. The presence of PUFAs can explain the reduced plasmatic levels of total cholesterol as they act as a protective factor in the homeostasis of cholesterol due to the high number of unsaturations and thus less phospholipid–cholesterol interaction [45].

Another study states that diets rich in MUFAs can reduce the levels of total cholesterol total and LDL-c [46]. This could be one of the factors contributing to the low levels of LDL-c and non-HDL-c we found, despite high levels of SFA associated with the high plasmatic levels of LDL-c [47]. The group supplemented with olive oil in higher dosage (2000 mg/kg) also showed values significantly reduced, possibly due to the higher concentration of MUFAs present in this oil, as well as in C. *brasiliense* oil, which are mainly composed of oleic acid, followed by palmitic and linoleic acid [48]. We point out that the supplementation with *C. brasiliense* oil (2000 mg/kg) reached a better response in the parameters that are commonly associated with atherosclerosis (LDL-c and non-HDL-c) [49] when compared with groups CG and OO1, supplemented with soybean and olive oils, respectively.

Regard to body weight gain and adiposity index (Table 4), we noticed that animals kept a pattern of weight not differing statistically (*p* ≤ 0.05). Nevertheless, the group receiving higher doses of MUFA (OO + *C. brasiliense* oil) presented the lowest mean weight. Similar results we observed in adipose tissue weight (Table 4), the group supplemented with olive oil plus *C. brasiliense* oil had lower weight of the principal visceral fats, including epididymal adipose tissue; in mice, this is one of the main deposit areas of visceral fat [50]. Other relevant sites, such as mesenteric and retroperitoneal fat [51], also diminished compared with the control group.

This effect can be associated with the presence of tocopherols and mainly of tocotrienols that are present in both *C. brasiliense* oil, as demonstrated in our study, and olive oil [52]. Another work pointed out that the ingestion of gamma-tocotrienol (60 mg/kg/day) was capable of reducing the fat mass induced by different doses of glucocorticoids. Uto-Kondo et al. (2009) [53] evaluated the effect of a palm oil fraction rich in tocotrienol on the differentiation of adipocyte into 3T3-L1 cells and found that this antioxidant suppressed the differentiation of pre-adipocytes into adipocytes, potentially reducing weight gain.

Among the possible alterations in the liver (Table 5), we did not identify statistical difference regarding the presence of hepatic steatosis (*p* = 0.17) and microvesicular steatosis (*p* = 0.45). However, a build-up of free FAs occurred in the liver, except group OO + CO that showed only microvesicular steatosis, demonstrating that the synergic effect of the mix of olive oil with *C. brasiliense* oil may have played a slight protective role, due to a higher concentration of antioxidants. These are known for beneficial action in biological systems and protection against oxidative damages [54] as the oxidative stress is one of the causes of hepatocellular lesions [51].

We detected significant differences between groups regarding the presence of Mallory Hyaline, and in post-test it was significantly more present in animals of all groups compared with GC, as well as group OO + CO compared with OO1. Apoptosis was significantly more prevalent in group CO1 compared with CG and OO1, and no presence was recorded in OO1. Palmeira et al. (2015) [55] reported that the administration of *C. brasiliense* oil at 400 mg/kg in mice induced with diethylnitrosamine 10 μg/g reduced the development of preneoplastic lesions and hepatic adenoma. Another study on *C. brasiliense* nut oil found that it can attenuate the biochemical markers of hepatic lesion and inflammation [21].

Among the data obtained in the histological analyses of the pancreas (Table 6), we did not find an association between the presence of Langerhans Islets (*p* = 0.93) or inflammatory cells in all groups (*p* = 0.38). None of the samples analyzed in our study showed alterations in pancreatic acini.

**Figure 5:**
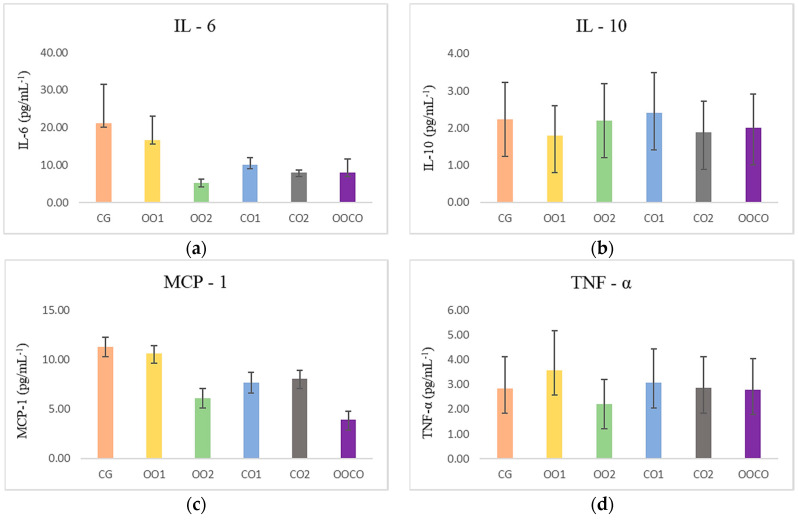
Effects of supplementation from different lipid source on anti- and proinflammatory cytokines. (**a**) Interleukin-6; (**b**) Interleukin-10; (**c**) Monocyte-1 chemotactic protein; (**d**) Tumor necrosis factor alpha. CG indicates control group, supplemented with soybean oil (1000 mg/kg); OO1 and OO2 groups receiving olive oil (1000 mg/kg and 2000 mg/kg, respectively); CO1 and CO2 groups supplemented with *Caryocar brasiliense* oil (1000 mg/kg and 2000 mg/kg, respectively); and OO + CO group receiving olive oil (1000 mg/kg) with *C. brasiliense* oil (1000 mg/kg). ANOVA: one-way analysis of variance.

The amount of consumed SFA influences the accumulation of free FAs in the liver and activation of inflammatory markers [56]. For the inflammatory response to start it needs proinflammatory cytokines and chemokines such as TNF-α, IL-6, and MCP-1 [57]. Figure 5 shows the levels of circulating inflammatory cytokines IL-6 (*p* = 0.944), MCP-1 6 (*p* = 0.640), TNF-α (*p* = 0.834), and anti-inflammatory IL-10 (*p* = 0.709), without significant difference between treatments. Recent studies reported that phytochemicals present in plants could inhibit the inflammation, reducing the production of macrophages, proinflammatory factors and also blocking inflammatory pathways that liberate cytokines [58,59]. In a study on rats utilizing supplementation with *C. brasiliense* nut oil at a concentration of 6 mL/kg, the results suggest that it attenuates the acute inflammatory response when induced by CCl_4_, modulating the circulating levels of leptin, IL-6, LTB4, and LTB-5 positively [21].

The tested dosages of CO did not demonstrate a protective effect on those parameters when compared with the group supplemented with soybean oil (CG) and both doses of olive oil (OO1; OO2). The cytokine IL-10 is necessary to inhibit the synthesis of proinflammatory cytokines [56]. It can exert anti-inflammatory effects through signal transducer pathway of the Janus kinase (JAK) of activation 3 (JAK-STAT3), binding IL-10 to the receptor in the targeted cell membrane—tyrosine kinase 2—leading to the activation of the signal transducer and activator of transcription 3 (STAT3). In our study, IL-10 was more active in the group supplemented with olive oil at the lower dose (1000 mg/kg). Nevertheless, more research is necessary to elucidate the molecular action mechanisms of oleic acid [21], which is the major component in CO and OO, and the phytochemicals present in *C. brasiliense* oil, such as phenolics, carotenoids, tocopherols, and phytosterols, that constitute possible mediators of these effects.

## 3. Materials and Methods

### 3.1. Raw Material

*Caryocar brasiliense* Cambess. Pulp oil was provided by RTK^®^ Cosmética e Indústria de Alimentos Naturais (Brasília, Brazil) and it had been extracted and cold-pressed. The soybean oil and olive oil were acquired from local shops.

### 3.2. Quality and Identity of C. brasiliense Oil

We qualified the oil regard to indexes of acidity (method Ca 5a-40), peroxide (method Cd 8-53), refraction (method Cc 7-25), iodine (method Cd 1-25), and saponification (method Cd 3-25) [60]. All analyzes were performed in triplicate. The acidity index (AI) was determined with 2 g of sample by the addition of a solution of ether-alcohol, using an indicator (phenolphthalein) and titration with NaOH until appearing the light pink color; the results of acidity in oleic acid were expressed in milligrams of sodium hydroxide per gram. The determination of the peroxide index (PI) was performed with 5 g of sample, in a solution of acetic acid-chloroform and potassium iodide, with posterior rest in the dark, titration with sodium thiosulfate 0.01 N, and addition of soluble starch as an indicator for observation of color change; the results were expressed in milliequivalents of peroxide per 1000 g of sample. The refraction index (RI) was read after filtering the sample to remove any impurities and traces of moisture, using a refractometer Abbé calibrated with distilled water (refraction index 1.3330) at 20 °C, with temperature corrected to 40 °C. For the iodine index (II), approximately 0.25 g of the sample of *C. brasiliense* oil was placed in Erlenmeyers with carbon tetrachloride and Wijs solution. Sodium thiosulfate was used for titration until the color changed from dark blue to pinkish, and the results were indicated in grams of iodine absorbed per gram of sample. The saponification index (SI) was determined by the addition of KOH and phenolphthalein to 5 g of the samples, and titration with HCl until pink disappeared and the value was expressed as the number of milligrams of potassium hydroxide (KOH) required to saponify 1 g of the oil sample.

#### 3.2.1. Color

We measured the oil color using a colorimeter (CM-2300d, Konica Minolta, Ramsey, NJ, EUA), expressed in the classification scale CIE-L*, a*, b*, where the values L* indicate the lightness, a* represents the red-green axis, and b* yellow-blue axis. From the obtained results, we determined the hue angle (h) and the chroma (C*).

#### 3.2.2. Total Carotenoids

For analysis of carotenoids, we utilized procedures described by Maldonade et al. (2012) [61] with slight modifications. Samples of 5 g *C. brasiliense* oil were placed in Erlenmeyer of 125 mL, we then added 25 mL acetone, shaked the solution, and stored it for two hours in the fridge. After centrifugation at 4000 rpm and 4 °C, the carotenoids were extracted by partitioning of the sobrenatant in petroleum ether, with successive washings with distilled water, followed by saponification with NaOH 10% in methylic alcohol for 2 h in the dark. The samples were again centrifuged and extracted by partitioning in petroleum ether, washed with distilled water, and recovered in a volumetric balloon, after excess water removal with anhydrous sodium sulfate. We made readings of absorbance in a spectrophotometer at 450 nm for quantification of total carotenoids.

#### 3.2.3. Optical Properties

The samples of *C. brasiliense* oil were diluted in hexane (spectroscopic grade 99.9%) at concentrations of 5 g/L. For optical measurements, we utilized a quartz cuvette with an optical path of 10 mm and four polished slides. We measured UV-Vis absorption using a spectrometer Lambda 265-Perkin Elmer^™^, Waltham, MA, USA and the absorption spectrum between 200 and 600 nm was collected. For the fluorescence map (excitation/emission), we used a bench-top spectrophotometer FS-2 (Scinco^™^, Seoul, Korea), the samples were excited from 200 to 400 nm every 5 nm, the measured emission being 250 and 600 nm with a 1 nm resolution. For all assessments, the excitation and emission slots were fixed in 5 nm. All optical measurements were performed at room temperature.

#### 3.2.4. Thermal Analyses: Thermogravimetry/Derived Thermogravimetry (TGA/DTG)

We performed the oil analyses on approximately 4 mg of sample in a thermal analyzer system (TGA Q50, TA Instruments, New Castle, DE, EUA), under an inert atmosphere of nitrogen with a flux of 60 mL/min^−1^, at a heating rate of 10 °C/min^−1^ with temperatures of 25 °C to 900 °C, utilizing platinum crucibles.

#### 3.2.5. Oxidative Stability

The oxidative stability was analyzed by measuring the induction period using the Rancimat method. We utilized the equipment 893 Professional Biodiesel Rancimat (Metrohm^®^, São Paulo, Brazil), where 3.0 g sample of the oil without dilution was put into a sealed glass reaction vessel and was submitted to 110 °C under constant air flux at 10 L h^−1^, which passed through the samples and then into a measuring vessel containing 50 mL ultrapure water in which the conductivity generated by volatile products during the vegetable oil degradation was measured as a function of time, according to the European rule EN14112. The induction period was determined by the second derivative method of the conductivity curve.

#### 3.2.6. Profile of Fatty Acids

The methylic esters of the fatty acids (FAME) were prepared according to the method of Maya and Rodriguez-Amaya with a solution of derivatization of ammonium chloride, methanol, and sulfuric acid. FAMEs were analyzed by gas chromatography (GC 2010, Shimadzu) to obtain their peaks. The equipment utilized a flame ionization detector (FID) and a capillary column (BPX-70, 0.25 m internal diameter, 30 mm long, and 0.25 mm thick). The temperature of the injector and the detector was 250 °C. The initial temperature of the column was 80 °C, which was held for 3 min and then increased at a rate of 10 °C min^−1^ until reaching 140 °C, followed by an increase to 240 °C at a rate of 5 °C min^−1^ for 5 min. We identified the individual peaks of FAME were identified comparing their relative retention time with the standard of 37 FAMEs (Supelco C22, 99 % pure).

### 3.3. Experimental Design

The project was carried out according to the ethic regulations and guidelines, and the experimental protocol approved by the Ethics Committee in Use of Animals (Protocol n. 954/2018). We utilized Swiss mice (*Mus musculus*), adult males, provided by the Central Biotherium/CCBS/UFMS, kept under temperature at 22 ± 2 °C, relative air humidity of 50–60%, with a light/dark cycle of 12 h, with standard diet AIN-93M and water ad libitum.

**Figure 6:**
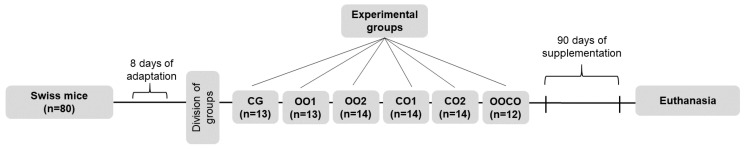
Intervention protocol of the supplementation with *Caryocar brasiliense* oil. CG indicates control group, supplemented with soybean oil (1000 mg/kg); OO1 and OO2 groups receiving olive oil (1000 mg/kg and 2000 mg/kg, respectively); CO1 and CO2 groups supplemented with *C. brasiliense* oil (1000 mg/kg and 2000 mg/kg, respectively); and OO + CO group receiving olive oil (1000 mg/kg) with *C. brasiliense* oil (1000 mg/kg).

The experimental protocol is shown in Figure 6. The mice (n = 80) were randomized into six treatment groups. After an 8-day adaptation period, we made supplementation for 90 days, with different lipidic sources (soybean oil, olive oil, and *C. brasiliense* pulp oil), via gavage, at different doses adjusted weekly, according to animal weight. The Control Group (CG) (n = 13) received Soybean Oil (1000 mg/kg); Group Olive Oil 1 (OO1) (n = 13) and Group Olive Oil 2 (OO2) (n = 14) were supplemented with extra-virgin olive oil (1000 mg/kg and 2000 mg/kg, respectively); Group *C. brasiliense* Oil 1 (CO1) (n = 14); Group *C. brasiliense* Oil 2 (CO2) (n = 14), received *C. brasiliense* oil (1000 mg/kg and 2000 mg/kg, respectively); and tGroup Olive Oil + *C. brasiliense* oil (OOCO) (n = 12), containing olive oil (1000 mg/kg) associated with *C. brasiliense* oil (1000 mg/kg). Euthanasia occurred by exsanguination by cardiac puncture, utilizing isoflurane as an anesthetic.

#### 3.3.1. Biochemical Analysis

Blood samples were collected and centrifuged to obtain the serum fractions and determine the levels of triacylglycerols, total cholesterol, LDL-c, HDL-c, VLDL-c, non-HDL-c, and blood glucose. The parameters were determined by the enzymatic-colorimetric method, according to the manufacturer’s instructions (Labtest™, Lagoa Santa, Minas Gerais, Brazil).

#### 3.3.2. Body Weight, Visceral Fat, and Liver Weight

The bodyweight of the animals was recorded weekly on a semi-analytic balance (Bel Diagnóstica^®^), expressed in grams. The liver and the sites of omental, epididymal, retroperitoneal, perirenal, and mesenteric fat of each animal were removed and weighed on a semi-analytic balance (Bel Diagnóstica^®^) for comparison between the studied groups. The adiposity index (AI) was calculated according to Equation (1) [62].

(1) $$\mathrm{AI}\left(\%\right)=\frac{\mathrm{Total}\;\mathrm{weight}\;\mathrm{of}\;\mathrm{white}\;\mathrm{visceral}\;\mathrm{fat}\;(g)\;}{\mathrm{Animal}\;\mathrm{final}\;\mathrm{body}\;\mathrm{weight}\;(g)}\;\times \;100$$

#### 3.3.3. Histopathological Analysis

Samples of liver and pancreas were fixed with 10% formalin solution. After fixation, the specimens were dehydrated, embedded in paraffin, cut in a microtome to a thickness of 5 mm each, and stained with hematoxylin and eosin [63,64].

Histological analysis was performed for an expert pathologist. For the analyses of effects of treatment on hepatocytes, we utilized the system of Kleiner et al. (2005) [63], evaluating the degree of steatosis (<5%, 5 to 33%, 34 to 66%, >66%), microvesicular steatosis (absent or present), lobular inflammation (Absent, <1 focus/field, 2–4 foci/field, or >4 foci/field), ballooning (absent, few cells, or many cells), Mallory hyaline (absent or present), glycogenated nuclei (none/rare or some), and apoptosis (absent or present).

We evaluated the architecture of the pancreas according to alterations in the Langerhans islets (without alteration, discrete atrophy, atrophy, discrete hypertrophy, and hypertrophy), pancreatic acini (without alteration, necrosis/atrophy), and inflammation by the presence of inflammatory cells inside (insulitis) and on the periphery (perinsulitis) in the Langerhans islets [65,66,67].

#### 3.3.4. Quantification of Cytokines

The serum was collected after centrifugation and stored again at −80 °C until cytokine analysis, according to the recommendations of the manufacturer (MILLIPLEX MAP/Mouse Cytokine/Chemokine and Adipocyte Magnetic Bead panel) (Millipore, Billerica, MA, USA). The concentrations of the following cytokines were analyzed: IL-10, IL-6, MCP-1, and TNF-α using the MCYTOMAG-70K kit, in MAGPIX^™^ with xPONENT software. The concentration of the cytokines IL-10, IL-6, MCP-1, and TNF-α in the serum was expressed as cytokine picograms related to protein content (mg of protein).

### 3.4. Statistical Analyses

The results were expressed as mean ± standard error. For multiple comparisons of parametric results, we performed an ANOVA followed by a Tukey post-test. The chi-square test was applied to evaluate associations in histological analyses, followed by a Bonferroni test. A significance level of *p* < 0.05 was adopted. For statistical analyses we used the software Jandel Sigma Stat, version 3.5 (Systat software, Incs., San Jose, CA, USA), and Bioestat 5.0 (Systat software, Incs., San Jose, CA, USA).

## 4. Conclusions

*Caryocar brasiliense* oil can be considered an oil with adequate quality for human consumption, with excellent oxidative stability and shelf-life, having the potential to be commercialized at large-scale in the food market. The *C. brasiliense* oil promotes beneficial effects on biochemical parameters, mainly in the lipidic profile, reducing indices associated with inflammatory processes. Combined with olive oil, *C. brasiliense* oil reduces the development of white adipose tissue, possibly due to the presence of antioxidants, such as tocopherols and tocotrienols.

## Figures and Tables

**Table 1:** Indices of quality and identity of *Caryocar brasiliense* pulp oil.

Index	Values
Peroxide index (mEq O_2_ kg^−1^)	13.63 ± 0.97
Acidity index in oleic acid (mg KOH/g^−1^)	1.27 ± 0.02
Saponification index (mg KOH g^−1^)	136.5 ± 0.60
Refraction index at 40 °C	1.46 ± 0.00
Iodine index (g I_2_ 100^−1^ g)	76.7 ± 0.72
Relative density	0.57 ± 0.00
L*	36.6 ± 0.03
C*	22.2 ± 0.02
h (deg)	74.3 ± 0.04
a*	6.0 ± 0.02
b*	21.4 ± 0.05
Carotenoids (µg/g)	2.39 ± 0.04

The results are expressed as the means ± mean standard error.

**Table 2:** Fatty acids profile of *Caryocar brasiliense* pulp oil (% in area).

Fatty Acids	Values
*Saturated*	
Butyric. C4:0	0.04 ± 0.01
Mystiric. C14:0	0.08 ± 0.00
Palmitic. C16:0	37.78 ± 1.07
Heptadecanoic. C17:0	0.05 ± 0.00
Stearic. C18:0	1.84 ± 0.00
Arachidic. C20:0	0.19 ± 0.02
Behenic. C22:0	0.06 ± 0.01
Lignoceric. C24:0	0.07 ± 0.01
TOTAL	40.04
*Monounsaturated*	
Palmitoleic. C16:1	0.57 ± 0.01
Oleic. C18:1 (𝜔-9)	52.61 ± 1.06
Cis-11- eicosenic. C20:1	0.19 ± 0.00
TOTAL	53.56
*Polyunsaturated*	
Linoleic. C18:2 (𝜔-6)	3.9 ± 0.15
0.40 ± 0.00	
0.04 ± 0.00	
TOTAL	4.34

The results are expressed as the means ± mean standard error.

**Table 3:** Biochemical parameters of animals after 90 days of *Caryocar brasiliense* oil supplementation.

Parameters (mg dL^−1^)	CG	OO1	OO2	CO1	CO2	OOCO
Total cholesterol	188.79 ± 9.18	177.68 ± 9.59	163.22 ± 10.80	134.61 ± 5.29 *^,§^	138.90 ± 6.01 *^,§^	149.85 ± 5.15 *
HDL-c	120.93 ± 5.18	119.62 ± 7.48	118.54 ± 6.37	97.26 ± 4.08	109.33 ± 6.13	111.75 ± 5.74
LDL-c	43.88 ± 5.02	33.36 ± 6.25	19.65 ± 6.05 *	14.10 ± 1.42 *	6.58 ± 1.88 *^,§^	15.47 ± 2.60 *
Non-HDL-c	69.10 ± 5.90	58.05 ± 6.46	44.68 ± 6.77	37.35 ± 1.88 *	29.57 ± 1.83 *^,§^	38.08 ± 2.91 *
VLDL-c	23.97 ± 4.18	24.70 ± 3.06	25.03 ± 3.31	23.25 ± 3.95	22.99 ± 2.35	22.62 ± 2.67
Triglycerides	188.77 ± 5.80	123.49 ± 4.43	125.16 ± 4.26	116.25 ± 5.27	114.97 ± 2.72	113.14 ± 3.86
Glucose	180.64 ± 21.50	179.12 ± 16.78	234.46 ± 13.19	216.68 ± 14.47	191.90 ± 10.33	229.29 ± 13.89

CG indicates control group, supplemented with soybean oil (1000 mg/kg); OO1 and OO2 groups receiving olive oil (1000 mg/kg and 2000 mg/kg, respectively); CO1 and CO2 groups supplemented with *C. brasiliense* oil (1000 mg/kg and 2000 mg/kg, respectively); and OO + CO group receiving olive oil (1000 mg/kg) with *C. brasiliense* oil (10 *C. brasiliense* oil 00 mg/kg). Values represent the mean ± mean standard error. * *p* ≤ 0.05 vs. CG; ^§^ *p* ≤ 0.05 vs. OO1. ANOVA: one-way analysis of variance with Tukey post hoc test.

**Table 4:** Bodyweight, liver, and visceral fats weight (g) of animals supplemented with different lipid sources.

Parameters	CG	OO1	OO2	CO1	CO2	OOCO
Initial weight (g)	39.231 ± 1.277	37.692 ± 1.157	40.286 ± 1.197	39.429 ± 1.561	38.500 ± 1.185	39.333 ± 0.873
Final weight (g)	48.923 ± 1.916	48.615 ± 1.591	52.714 ± 1.535	51.286 ± 1.871	47.429 ± 1.349	46.333 ± 2.028
Omental weight (g)	0.073 ± 0.011	0.050 ± 0.009	0.053 ± 0.006	0.038 ± 0.007 *	0.031 ± 0.006 *	0.026 ± 0.004 *
Epididymal weight (g)	1.970 ± 0.224	1.817 ± 0.123	1.715 ± 0.175	1.583 ± 0.177	1.281 ± 0.134 *	1.055 ± 0.132 *^,§^
Mesenteric weight (g)	1.008 ± 0.110	0.970 ± 0.095	1.083 ± 0.164	0.847 ± 0.107	0.850 ± 0.115	0.528 ± 0.074 *^,§,¥^
Retroperitoneal weight (g)	0.693 ± 0.088	0.670 ± 0.065	0.488 ± 0.064	0.462 ± 0.066	0.363 ± 0.047 *^,§^	0.330 ± 0.036 *^,§^
Perirenal weight (g)	0.391 ± 0.046	0.368 ± 0.047	0.304 ± 0.050	0.212 ± 0.030 *	0.209 ± 0.031 *	0.151 ± 0.017 *^,§^
Adiposity index (%)	8.290 ± 0.620	7.888 ± 0.378 *	6.799 ± 0.632 *	5.986 ± 0.567	5.640 ± 0.515 ^§^	4.422 ± 0.436 *^,§,¥^
Liver (g)	1.70 ± 0.086	1.66 ± 0.070	1.55 ± 0.118	1.43 ± 0.093	1.33 ± 0.035 *^,§^	1.40 ± 0.046

CG indicates control group, supplemented with soybean oil (1000 mg/kg); OO1 and OO2 groups receiving olive oil (1000 mg/kg and 2000 mg/kg, respectively); CO1 and CO2 groups supplemented with *Caryocar brasiliense* oil (1000 mg/kg and 2000 mg/kg, respectively); and OO + CO group receiving olive oil (1000 mg/kg) with *C. brasiliense* oil (1000 mg/kg). Values represent the mean ± mean standard error. * *p* ≤ 0.05 vs. CG; ^§^ *p* ≤ 0.05 vs. OO1; ^¥^ *p* ≤ 0.05 vs. OO2. ANOVA: one-way analysis of variance with Tukey post hoc test.

**Table 5:** Distribution of changes observed in the liver of the animals in the experimental groups.

Variable	CG	OO1	OO2	CO1	CO2	OOCO
Steatosis (*p* = 0.17 ^A^)						
<5%	36.36 (4)	60.0 (6)	80.0 (8)	80.0 (8)	40.0 (4)	66.7 (6)
5–33%	18.18 (2)	20.0 (2)	10.0 (1)	10.0 (1)	40.0 (4)	33.3 (3)
33–66%	45.45 (5)	20.0 (2)	10.0 (1)	10.0 (1)	20.0 (2)	0.0 (0)
Microvesicular Steatosis (*p* = 0.45)						
Absent	81.8 (9)	90.0 (9)	100.0 (10)	90.0 (9)	100.0 (10)	88.9 (8)
Present	18.2 (2)	10.0 (1)	0.0 (0)	10.0 (1)	0.0 (0)	11.1 (1)
Lobular Inflammation (*p* = 0.16 ^B^)						
Absent	54.6 (6)	70.0 (7)	50.0 (5)	50.0 (5)	20.0 (2)	77.8 (7)
<2 focus	45.4 (5)	30.0 (3)	50.0 (5)	50.0 (5)	70.0 (7)	22.2 (2)
2–4 focuses	0.0 (0)	0.0 (0)	0.0 (0)	0.0 (0)	10.0 (1)	0.0 (0)
Ballooning (*p* = 0.06 ^B^)						
Absent	72.7 (8)	80.0 (8)	20.0 (2)	0.0 (0)	10.0 (1)	0.0 (0)
Few cells	27.3 (3)	20.0 (2)	20.0 (2)	30.0 (3)	30.0 (3)	11.1 (1)
Many cells	0.0 (0)	0.0 (0)	40.0 (6)	70.0 (7)	60.0 (6)	88.9 (8)
Mallory’s Hyaline (*p* < 0.001 *^,B^)						
Absent	100.0 (11)	80.0 (8)	40.0 (4)	20.0 (2)	40.0 (4)	11.1 (1)
Rare	0.0 (0)	20.0 (2)	40.0 (4)	40.0 (4)	40.0 (4)	66.7 (6)
Some	0.0 (0)	0.0 (0)	20.0 (2)	40.0 (4)	20.0 (2)	22.2 (2)
Apoptosis (*p* = 0.001 *)						
Absent	81.8 (9)	100.0 (10)	70.0 (7)	20.0 (2)	30.0 (3)	44.4 (4)
Present	18.2 (2)	0.0 (0)	30.0 (3)	80.0 (8)	70.0 (7)	55.6 (5)
Glycogenated Nuclei (*p* = 0.07)						
None/rare	100.0 (11)	90.0 (9)	100.0 (10)	90.0 (9)	100.0 (10)	88.9 (8)
Some	0.0 (0)	10.0 (1)	0.0 (0)	10.0 (1)	0.0 (0)	11.1 (1)

CG indicates control group, supplemented with soybean oil (1000 mg/kg); OO1 and OO2 groups receiving olive oil (1000 mg/kg and 2000 mg/kg, respectively); CO1 and CO2 groups supplemented with *Caryocar brasiliense* oil (1000 mg/kg and 2000 mg/kg, respectively); and OO + CO group receiving olive oil (1000 mg/kg) with *C. brasiliense* oil (1000 mg/kg). Data are presented in relative frequency (absolute frequency). *p*-value in the chi-square test. Due to the sample size, two categories were grouped as follows: A ≤ 5% and ≥5%; B = Absent and Present, * *p* ≤ 0.05.

**Table 6:** Distribution of changes observed in animal pancreas in each experimental group.

Variable	Experimental Group % (n)					
	CG	OO1	OO2	CO1	CO2	OOCO
Islet of Langerhans (*p* = 93 ^A^)						
No change	45.4 (5)	33.33 (3)	36.4 (4)	33.33 (3)	45.4 (5)	36.4 (4)
Discrete atrophy	36.4 (4)	66.7 (6)	33.33 (3)	33.33 (3)	33.33 (3)	33.33 (3)
Atrophy	0.0 (0)	0.0 (0)	22.2 (2)	33.3 (3)	11.1 (1)	0.0 (0)
Hypertrophy	9.1 (1)	0.0 (0)	0.0 (0)	0.0 (0)	0.0 (0)	22.2 (2)
Not available	9.1 (1)	0.0 (0)	0.0 (0)	0.0 (0)	0.0 (0)	0.0 (0)
Pancreatic Acini (^B^)						
No change	100.0 (11)	100.0 (9)	100.0 (9)	100.0 (9)	100.0 (9)	100.0 (9)
Inflammatory Cells (*p* = 0.38)						
No change	100.0 (11)	100.0 (9)	88.9 (8)	100.0 (9)	100.0 (9)	100.0 (9)
Insulits	0.0 (0)	0.0 (0)	11.1 (1)	0.0 (0)	0.0 (0)	0.0 (0)

CG indicates control group, supplemented with soybean oil (1000 mg/kg); OO1 and OO2 groups receiving olive oil (1000 mg/kg and 2000 mg/kg, respectively); PO1 and PO2 groups supplemented with *Caryocar brasiliense* oil (1000 mg/kg and 2000 mg/kg, respectively); and OO + CO group receiving olive oil (1000 mg/kg) with *C. brasiliense* oil (1000 mg/kg). Data are presented in relative frequency (absolute frequency). *p*-value in the chi-square test. (A) Due to the sample size, the categories were grouped into No change and with change; (B) Inferential statistical analysis due to the absence of variability between groups.
